# Effects of Maternal Subclinical Hypothyroidism in Early Pregnancy Diagnosed by Different Criteria on Adverse Perinatal Outcomes in Chinese Women With Negative TPOAb

**DOI:** 10.3389/fendo.2020.580380

**Published:** 2020-10-08

**Authors:** Mei-Fang Li, Li Ma, Qi-Ming Feng, Yue Zhu, Tian-Pei Yu, Jiang-Feng Ke, Zhi-Hui Zhang, Yun Liu, Lian-Xi Li

**Affiliations:** ^1^Department of Emergency, Shanghai JiaoTong University Affiliated Sixth People's Hospital, Shanghai, China; ^2^Department of Obstetrics and Gynecology, Shanghai Clinical Center for Severe Maternal Rescue, Shanghai JiaoTong University Affiliated Sixth People's Hospital, Shanghai, China; ^3^Department of Endocrinology and Metabolism, Shanghai Jiao Tong University Affiliated Sixth People's Hospital, Shanghai Clinical Center for Diabetes, Shanghai Diabetes Institute, Shanghai Key Laboratory of Diabetes Mellitus, Shanghai Key Clinical Center for Metabolic Disease, Shanghai, China; ^4^Department of Information, Department of Medical Information, School of Biomedical Engineering and Informatics, The First Affiliated Hospital of Nanjing Medical University, Nanjing Medical University, Jiangsu, China

**Keywords:** subclinical hypothyroidism, pregnancy, adverse maternal outcomes, adverse neonatal outcomes, guidelines of the American thyroid association

## Abstract

**Aims:** To compare the effects of maternal subclinical hypothyroidism (SCH) diagnosed by the 2011 or 2017 “Guidelines of the American Thyroid Association (ATA) for the diagnosis and management of thyroid disease during pregnancy and the postpartum” during the first trimester on adverse pregnancy outcomes in thyroid peroxidase antibody (TPOAb)–negative pregnant women.

**Methods:** There were 1,556 Chinese singleton pregnant women with negative TPOAb diagnosed with either SCH or euthyroidism who were investigated, and the prevalence and risk of obstetric outcomes were compared between the two groups using 2011 and 2017 ATA standards, respectively. The effects of a mildly elevated thyroid-stimulating hormone (TSH) concentration on adverse pregnancy outcomes were evaluated by binary logistic regression.

**Results:** Maternal SCH identified by the 2011 ATA guidelines correlated with higher rates and risks of pregnancy-induced hypertension (PIH), preeclampsia, and low-birth-weight infants, while maternal SCH diagnosed by the 2017 ATA guidelines was more likely to develop PIH, preeclampsia, cesarean delivery, preterm delivery, placenta previa, and total adverse maternal and neonatal outcomes. Moreover, a mildly elevated TSH level was significantly associated with PIH after adjustment for confounding factors.

**Conclusions:** Compared with the 2011 ATA guidelines, the 2017 ATA guidelines could be more applicable to Chinese pregnant women to screen the effects of SCH on the majority of adverse pregnancy outcomes.

## Introduction

Despite the well-known harmful effects of overt hypothyroidism on pregnancy outcomes in women at childbearing ages (1–3), the impacts of subclinical hypothyroidism (SCH), characterized by an elevated thyroid-stimulating hormone (TSH) with normal thyroxine (fT4), on adverse obstetric outcomes have not been yet clearly identified. Some studies indicated that SCH was associated with several obstetric complications, including preeclampsia, preterm delivery, and placental abruption (4–6), whereas others revealed that SCH did not result in poor pregnancy outcomes (7, 8) These inconsistencies may be mainly attributed to the differences of the diagnostic criteria for SCH (different TSH cutoffs) in different studies. Therefore, how to define SCH in pregnancy has been increasingly debated in recent years.

The 2011 “Guidelines of the American Thyroid Association (ATA) for the Diagnosis and Treatment of Thyroid Disease During Pregnancy and the Postpartum” recommended that the ideal upper limit of serum TSH was 2.5 mIU/L in early pregnancy (9). However, in recent years, several scholars found that SCH may be overdiagnosed in a large proportion of pregnant, which may bring anxieties to pregnant woman and thus add their psychological stresses (10–12). Additionally, results regarding whether a mildly elevated TSH concentration could increase adverse pregnancy outcomes have been vigorously debated (8, 13, 14). Therefore, accordingly, a more liberal reference upper limit of TSH at 4.0 mIU/L in healthy pregnant women has been recommended by the 2017 ATA guidelines (15).

However, studies regarding comparisons between the effects of SCH diagnosed the 2011 and 2017 ATA guidelines on adverse perinatal outcomes are extremely limited. And there is still insufficient evidence on current studies whether the 2017 ATA guidelines are applicable to Chinese pregnant women. Therefore, one of the aims of our study was to compare and evaluate the influence of different maternal SCH, defined by the 2011 and 2017 ATA criteria, respectively, during the first trimester, on diversified adverse maternal and neonatal outcomes in Chinese pregnant women with negative anti–thyroperoxidase antibodies (TPOAbs). Furthermore, we also explored whether a mildly elevated TSH concentration during the first trimester could increase the risks of obstetric outcomes or not.

## Materials and Methods

### Study Population

It was a cross-sectional study with data from our previous study (16). Briefly, 4,178 pregnant women delivered at the obstetrics department of Shanghai JiaoTong University Affiliated Sixth People's Hospital from January to December in 2016 were collected as potential participants. First, 1,542 candidates without the thyroid function test results during 10–12 weeks, 373 candidates without oral glucose tolerance test (OGTT) results during 24–28 weeks, and 86 women with incomplete data were removed. Second, two candidates with reproductive system anatomy abnormalities and 92 candidates with known chronic and autoimmune diseases, such as preexisting diabetes and chronic hypertension, were also excluded. Third, for the remaining 2,083 cases, 527 candidates were successively eliminated based on the recent ATA guidelines (9, 17) that pregnant women with (1) known or clinically antepartum thyroid diseases and goiter, (2) current or previous treatment with antithyroid drugs or levothyroxine (LT4), (3) history of iodine-131 therapy or thyroidectomy, (4) use of medications that may influence thyroid function, (5) family history of thyroid diseases, (6) history of head and neck external radiotherapy, (7) TSH concentration less than the lower limit of the reference range provided by the test kit (0.27 mIU/L in this study) or higher than 10 mIU/L, (8) FT4 out of the reference range (12–22 pmol/L in this study), and (9) anti-TPOAb levels higher than the upper limit of the reference value (35 KIU/L in this study). Ultimately, 1,556 TPOAb-negative pregnant women joined in our study.

According to the 2011 and 2017 ATA criteria (9, 15), the subjects were divided into two groups including euthyroidism (ET) and SCH. In addition, to analyze the impact of a mildly elevated TSH concentration on adverse pregnancy outcomes, all patients were further stratified into three groups as maternal TSH values 0.27–2.5 mIU/L (the normal TSH group, *n* = 971), 2.5–4.0 mIU/L (the TSH mildly elevated group, *n* = 433), and 4.0–10 mIU/L (the TSH significantly elevated group, *n* = 152). Our study was approved by the ethics committee of the Shanghai JiaoTong University Affiliated Sixth People's Hospital, and written consent was obtained from the subjects individually.

### Data Collection

Details on the maternal age, prepregnancy's height and body weight, primiparity, number of pregnancies and previous births, gestational age, gestational weight gain, neonatal sex, birth weight and height, and Apgar scores, as well as adverse pregnancy outcomes, were obtained from their medical records. The pregestational body mass index (BMI) was equal to the weight divided by height squared (kg/m^2^).

### Laboratory Examinations

Blood sample was obtained from each participant after an overnight fast during 10–12 weeks. TSH, FT4, and TPOAb were measured by a chemiluminescence immunoassay (Roche Cobas 6000; Roche Ltd., Basel, Switzerland). A standard 75-g OGTT was performed at 24 to 28 weeks of gestation after an 8- to 10-h fast.

### Outcomes and Diagnostic Criteria

Both maternal and neonatal outcomes were obtained in our results. And pregnancy-induced hypertension (PIH), preeclampsia, GDM, cesarean delivery (CS), preterm delivery, postpartum hemorrhage, placental previa and abruption, and dystocia were shown in our maternal outcomes, and one or more of the above complications resulted in the total adverse maternal outcomes. Moreover, low birth weight, preterm birth, macrosomia, fetal distress, fetal asphyxia, fetal deformities, and stillbirth were exhibited in our neonatal outcomes, and one or more above characters generated the total adverse neonatal outcomes. The above definitions had already been defined in our early study [(16), Supplementary Table 1].

The 2011 ATA guidelines define SCH in pregnancy as TSH >2.5 mU/L with normal FT4 levels (9), and pregnant women with TSH >4.0 mU/L with normal FT4 levels were defined as SCH according to the 2017 ATA guidelines (15). In addition, the normal reference ranges of FT4 and anti-TPOAb levels provided by the test kit were 12–22 pmol/L and 35 KIU/L, respectively, in this study.

### Statistical Analyses

The data were analyzed using SPSS 19.0 software. Data were presented as either mean ± standard deviation or median with interquartile range or frequency following the below rules. First, normality was checked for continuous variables. And *t*-test was employed, and the variables were presented as mean ± standard deviation if the data showed a normal distribution, whereas the Mann-Whitney *U* test was used, and the variables were exhibited as median with interquartile range if not. Second, the χ^2^ test was employed, and variables were demonstrated by absolute numbers (percentages) on categorical variables. Moreover, binary logistic regression was further performed to assess the differences in categorical variables. *P* < 0.05 (two-sided) was considered to be statistically significant, whereas *P* < 0.10 (two-sided) was regarded as evident of statistical trends.

## Results

### Basic Characteristics of Pregnant Women and Their Offspring by Different SCH Standards

Of all the 1,556 enrolled women, the median age was 29 (27–33) years, and the median pregestational BMI was 21.48 (19.56–24.34) kg/m^2^. And the basic characteristics of pregnant women and their newborns by different SCH standards are shown in Table 1. According to the 2011 ATA criteria, 971 women were in the ET group, and 585 women were in the SCH group, and women diagnosed with SCH had significantly lower maternal age, prepregnancy BMI, and higher gestation age compared with those ET subjects (all *P* < 0.05). Based on the 2017 ATA criteria, there were 1,404 ET and 152 SCH pregnant women, and women identified with SCH in the early pregnancy had a lower prepregnancy BMI and neonatal birth weight and a higher gestation age compared with those with ET (all *P* < 0.05).

**Table 1:** Characteristics of pregnant women and their offspring by different SCH standards.

**Variables**	**ET**	**SCH**	***P* value**	***P*′ value**
**SCH diagnosed by 2011 standards**				
Number	971	585		
Maternal age (years)	30 ± 5	29 ± 4	0.008	—
Prepregnancy BMI (kg/m^2^)^a^	22.58 (18.73–25.39)	20.57 (17.95–22.59)	<0.001	<0.001
Number of pregnancies, n (%)			0.377	0.865
1	408 (42)	267 (45.6)		
2	283 (29.2)	160 (27.4)		
3+	280 (28.8)	158 (27)		
Number of previous births, n (%)			0.896	0.591
0	472 (48.6)	286 (48.9)		
1	479 (49.3)	285 (48.7)		
2+	20 (2.1)	14 (2.4)		
Primiparity, n (%)	472 (48.6)	286 (48.9)	0.915	0.401
Gestational age (weeks)^a^	38 (36–39)	39 (37–40)	<0.001	<0.001
Gestational weight gain (kg)	12.1 ± 4.1	12.0 ± 4.0	0.584	0.405
**Neonatal**				
Male, n (%)	461 (49.4)	311 (54)	0.084	0.082
Birth weight (g)	3,288 ± 459	3,275 ± 501	0.595	0.647
Birth height (cm)	49.7 ± 2.3	49.7 ± 1.4	0.761	0.789
Apgar scores	9.9 ± 1.0	9.9 ± 0.7	0.219	0.249
**SCH diagnosed by 2017 standards**				
Number	1,404	152		
Maternal age (years)	30 ± 4	29 ± 4	0.119	—
Prepregnancy BMI (kg/m^2^)^a^	21.64 (19.66–24.56)	20.29 (18.81–22.04)	<0.001	<0.001
Number of pregnancies, n (%)			0.571	0.790
1	603 (42.9)	72 (47.4)		
2	402 (28.6)	41 (27)		
3+	399 (28.5)	39 (25.6)		
Number of previous births, n (%)			0.961	0.824
0	685 (48.8)	73 (48)		
1	688 (49)	76 (50)		
2+	31 (2.2)	3 (2)		
Primiparity, n (%)	685 (48.8)	73 (48)	0.858	0.470
Gestational age (weeks)^a^	39 (36–39)	39 (38–40)	<0.001	0.045
Gestational weight gain (kg)	12.1 ± 4.1	12.0 ± 4.1	0.142	0.554
**Neonatal**				
Male, n (%)	684 (50.3)	88 (58.7)	0.053	0.053
Birth weight (g)	3,292 ± 468	3,206 ± 533	0.036	0.039
Birth height (cm)	49.7 ± 2.0	49.5 ± 2.1	0.184	0.158
Apgar scores	9.9 ± 0.9	9.9 ± 1.1	0.054	0.501

*ET, euthyroidism; SCH, subclinical hypothyroidism*.

*Continuous variables are expressed as mean ± SD or median with interquartile range, whereas categorical variables are expressed as percentages*.

a *Non-normal distribution of continuous variables*.

*P′value, adjusted for age*.

### Association of Maternal SCH and Adverse Pregnancy Outcomes by the 2011 ATA Standards

As shown in Table 2, based on the 2011 ATA guidelines, compared with the ET group, the prevalence and risks of PIH [3.8 vs. 1.5%, odds ratio (OR) = 4.27, 95% confidence interval (CI) = 1.95–9.33], preeclampsia (2.1 vs. 0.5%, OR = 7.40, 95% CI = 2.08–26.35), and low-birth-weight infants (4.3 vs. 2.7%, OR = 1.93, 95% CI = 1.02–3.62) were significantly higher in the SCH group after controlling for confounding factors. And a rising trend was observed in the SCH group for the risks of CS (36.9 vs. 36.4%, OR = 1.25, 95% CI = 0.98–1.59) and placenta previa (1.9 vs. 1.3%, OR = 2.21, 95% CI = 0.92–5.32) in contrast to the ET group. However, no demonstrable differences were found in the incidence of GDM, preterm delivery, postpartum hemorrhage, placenta abruption, dystocia, macrosomia, fetal distress, fetal asphyxia, fetal deformities, stillbirth, and total adverse maternal and neonatal outcomes.

**Table 2:** Association of maternal SCH and adverse pregnancy outcomes according to 2011 ATA standard.

**Outcomes**	**ET, n (%)**	**SCH2011, n (%)**	**Unadjusted OR (95% CI)**			**Model I OR (95% CI)**			**Model II OR (95% CI)**		
			**ET**	**SCH2011**	***P-*value**	**ET**	**SCH2011**	***P-*value**	**ET**	**SCH2011**	***P-*value**
PIH	15 (1.5%)	22 (3.8%)	1 (ref)	2.49 (1.28–4.84)	0.007	1 (ref)	3.86 (1.81–8.23)	<0.001	1 (ref)	4.27 (1.95–9.33)	<0.001
Preeclampsia	5 (0.5%)	12 (2.1%)	1 (ref)	4.05 (1.42–11.54)	0.009	1 (ref)	6.08 (1.82–20.28)	0.003	1 (ref)	7.40 (2.08–26.35)	0.002
GDM	194 (20%)	116 (19.8%)	1 (ref)	0.99 (0.77–1.28)	0.943	1 (ref)	1.09 (0.83–1.44)	0.524	1 (ref)	1.07 (0.81–1.41)	0.644
CS	353 (36.4%)	216 (36.9%)	1 (ref)	1.03 (0.83–1.27)	0.821	1 (ref)	1.21 (0.96–1.53)	0.115	1 (ref)	1.25 (0.98–1.59)	0.069
Preterm delivery	468 (48.2%)	277 (47.4%)	1 (ref)	0.97 (0.79–1.19)	0.746	1 (ref)	1.14 (0.91–1.43)	0.259	1 (ref)	1.15 (0.92–1.45)	0.223
Postpartum hemorrhage	12 (1.2%)	13 (2.2%)	1 (ref)	1.82 (0.82–4.01)	0.139	1 (ref)	1.48 (0.65–3.33)	0.348	1 (ref)	1.54 (0.68–3.52)	0.303
Placenta previa	13 (1.3%)	11 (1.9%)	1 (ref)	1.41 (0.63–3.17)	0.403	1 (ref)	2.31 (0.96–5.56)	0.062	1 (ref)	2.21 (0.92–5.32)	0.078
Placenta abruption	1 (0.1%)	1 (0.2%)	1 (ref)	1.66 (0.10–26.61)	0.720	1 (ref)	1.99 (0.11–36.51)	0.644	1 (ref)	1.94 (0.09–40.65)	0.670
Dystocia	14 (1.4%)	8 (1.4%)	1 (ref)	0.95 (0.40–2.27)	0.904	1 (ref)	0.94 (0.38–2.33)	0.891	1 (ref)	0.93 (0.38–2.29)	0.868
Low birth weight	26 (2.7%)	25 (4.3%)	1 (ref)	1.62 (0.93–2.84)	0.090	1 (ref)	2.00 (1.06–3.76)	0.032	1 (ref)	1.93 (1.02–3.62)	0.042
Preterm birth	468 (48.2%)	277 (47.4%)	1 (ref)	0.97 (0.79–1.19)	0.746	1 (ref)	1.14 (0.91–1.43)	0.223	1 (ref)	1.15 (0.92–1.45)	0.223
Macrosomia	47 (4.8%)	27 (4.6%)	1 (ref)	0.95 (0.59–1.55)	0.840	1 (ref)	0.94 (0.57–1.57)	0.823	1 (ref)	0.94 (0.56–1.58)	0.801
Fetal distress	17 (1.8%)	15 (2.6%)	1 (ref)	1.48 (0.73–2.98)	0.278	1 (ref)	1.68 (0.79–3.57)	0.179	1 (ref)	1.71 (0.80–3.69)	0.168
Fetal asphyxia	3 (0.3%)	2 (0.3%)	1 (ref)	1.11 (0.18–6.64)	0.912	1 (ref)	0.92 (0.08–10.11)	0.948	1 (ref)	0.86 (0.08–9.36)	0.903
Fetal deformities	12 (1.2%)	2 (0.3%)	1 (ref)	0.27 (0.06–1.23)	0.091	1 (ref)	0.74 (0.12–4.37)	0.736	1 (ref)	0.64 (0.08–4.78)	0.659
Stillbirth	9 (0.9%)	3 (0.5%)	1 (ref)	0.55 (0.15–2.04)	0.373	1 (ref)	0.48 (0.09–2.51)	0.387	1 (ref)	0.53 (0.10–2.96)	0.470
Total adverse	481 (49.5%)	290 (49.6%)	1 (ref)	1.00 (0.82–1.23)	0.989	1 (ref)	1.14 (0.91–1.43)	0.268	1 (ref)	1.17 (0.93–1.47)	0.177
maternal outcomes											
Total adverse	513 (52.8%)	299 (51.1%)	1 (ref)	0.93 (0.76–1.15)	0.510	1 (ref)	1.18 (0.94–1.48)	0.150	1 (ref)	1.18 (0.94–1.49)	0.155
neonatal outcomes											

*Model I, adjusted for maternal age, pregestational BMI, and gestation age; Model II, adjusted for maternal age, pregestational BMI, gestational age, number of previous pregnancies, number of previous births, primiparity, and gestational weight gain*.

### Association of Maternal SCH and Adverse Pregnancy Outcomes According to the 2017 ATA Standards

As depicted in Table 3, according to the 2017 ATA guidelines, pregnant women with SCH were more likely to have PIH (6.6 vs. 1.9%, OR = 5.16, 95% CI = 2.30–11.54), preeclampsia (5.3 vs. 0.6%, OR = 10.91, 95% CI = 3.72–32.01), CS (42.1 vs. 36%, OR = 1.68, 95% CI = 1.16–2.42), preterm delivery (53.9 vs. 47.2%, OR = 1.65, 95% CI = 1.16–2.37), and placenta previa (3.3 vs. 1.4%, OR = 4.12, 95% CI = 1.42–11.97) than those with ET, and their infants were more inclined to have lower birth weight (5.9 vs. 3%, OR = 2.19, 95% CI = 1.00–4.80) and experience fetal distress (3.9 vs. 1.9%, OR = 2.27, 95% CI = 0.89–5.78). Moreover, we observed that the prevalence and risks of total adverse maternal outcomes (69.1 vs. 62.5%, OR = 1.61, 95% CI = 1.10–2.34) and neonatal outcomes (58.6 vs. 51.5%, OR = 1.71, 95% CI = 1.19–2.44) in women identified with SCH displayed prominent rise compared with those with ET even after adjustment for relevant confounders. However, there were no significant differences in the prevalence of GDM, postpartum hemorrhage, placental abruption, dystocia, macrosomia, fetal asphyxia, fetal deformities, and stillbirth between the two groups.

**Table 3:** Association of maternal SCH and adverse pregnancy outcomes according to the 2017 ATA standard.

**Outcomes**	**ET, n (%)**	**SCH2017, n (%)**	**Unadjusted OR (95% CI)**			**Model I OR (95% CI)**			**Model II OR (95% CI)**		
			**ET**	**SCH2017**	***P-*value**	**ET**	**SCH2017**	***P-*value**	**ET**	**SCH2017**	***P-*value**
PIH	27 (1.9%)	10 (6.6%)	1 (ref)	3.59 (1.70–7.57)	0.001	1 (ref)	5.12 (2.31–11.38)	<0.001	1 (ref)	5.16 (2.30–11.54)	<0.001
Preeclampsia	9 (0.6%)	8 (5.3%)	1 (ref)	8.61 (3.27–22.66)	<0.001	1 (ref)	11.07 (3.80–32.24)	<0.001	1 (ref)	10.91 (3.72–32.01)	<0.001
GDM	277 (19.7%)	33 (21.7%)	1 (ref)	1.13 (0.75–1.70)	0.562	1 (ref)	1.21 (0.79–1.85)	0.376	1 (ref)	1.21 (0.79–1.85)	0.373
CS	505 (36%)	64 (42.1%)	1 (ref)	1.30 (0.92–1.82)	0.136	1 (ref)	1.62 (1.13–2.32)	0.009	1 (ref)	1.68 (1.16–2.42)	0.006
Preterm delivery	663 (47.2%)	82 (53.9%)	1 (ref)	1.31 (0.94–1.83)	0.116	1 (ref)	1.60 (1.12–2.29)	0.009	1 (ref)	1.65 (1.16–2.37)	0.006
Postpartum hemorrhage	22 (1.6%)	3 (2.0%)	1 (ref)	1.27 (0.37–4.28)	0.705	1 (ref)	1.08 (0.32–3.67)	0.907	1 (ref)	1.01 (0.29–3.48)	0.986
Placenta previa	19 (1.4%)	5 (3.3%)	1 (ref)	2.48 (0.91–6.74)	0.075	1 (ref)	3.81 (1.33–10.95)	0.013	1 (ref)	4.12 (1.42–11.97)	0.009
Placenta abruption	2 (0.1%)	0 (0%)	1 (ref)	—	0.996	1 (ref)	—	0.996	1 (ref)	—	0.996
Dystocia	19 (1.4%)	3 (2.0%)	1 (ref)	1.47 (0.43–5.02)	0.538	1 (ref)	1.45 (0.42–5.04)	0.560	1 (ref)	1.46 (0.42–5.09)	0.556
Low birth weight	42 (3%)	9 (5.9%)	1 (ref)	2.04 (0.97–4.28)	0.059	1 (ref)	2.25 (1.03–4.92)	0.042	1 (ref)	2.19 (1.00–4.80)	0.050
Preterm birth	663 (47.2%)	82 (53.9%)	1 (ref)	1.31 (0.94–1.83)	0.116	1 (ref)	1.60 (1.12–2.29)	0.009	1 (ref)	1.65 (1.16–2.37)	0.006
Macrosomia	68 (4.8%)	6 (3.9%)	1 (ref)	0.81 (0.34–1.89)	0.623	1 (ref)	0.82 (0.35–1.96)	0.662	1 (ref)	0.83 (0.35–2.00)	0.676
Fetal distress	26 (1.9%)	6 (3.9%)	1 (ref)	2.18 (0.88–5.37)	0.092	1 (ref)	2.32 (0.91–5.87)	0.077	1 (ref)	2.27 (0.89–5.78)	0.085
Fetal asphyxia	5 (0.4%)	0 (0%)	1 (ref)	—	0.996	1 (ref)	—	0.996	1 (ref)	—	0.996
Fetal deformities	14 (1.0%)	0 (0%)	1 (ref)	—	0.996	1 (ref)	—	0.996	1 (ref)	—	0.996
Stillbirth	10 (0.7%)	2 (1.3%)	1 (ref)	1.86 (0.40–8.56)	0.426	1 (ref)	1.48 (0.17–12.99)	0.724	1 (ref)	1.79 (0.20–16.12)	0.603
Total adverse	686 (48.9%)	85 (55.9%)	1 (ref)	1.33 (0.95–1.86)	0.099	1 (ref)	1.59 (1.09–2.31)	0.015	1 (ref)	1.61 (1.13–2.30)	0.009
maternal outcomes											
Total adverse	723 (51.5%)	89 (58.6%)	1 (ref)	1.33 (0.95–1.87)	0.099	1 (ref)	1.69 (1.18–2.42)	0.004	1 (ref)	1.71 (1.19–2.44)	0.004
maternal outcomes											

*Model I, adjusted for maternal age, pregestational BMI, and gestation age; Model II, adjusted for maternal age, pregestational BMI, gestational age, number of previous pregnancies, number of previous births, primiparity, and gestational weight gain*.

### Comparisons on the Basic Characteristics and Incidences of Adverse Pregnancy Outcomes Between SCH Defined by the 2011 and 2017 ATA Standards

The basic characteristics and incidences of maternal and neonatal outcomes are compared between SCH defined by the 2011 and 2017 ATA standards (Table 4). There was no difference in basic characteristics between the 2011 and 2017 ATA standards. The prevalence of preeclampsia in SCH women diagnosed by the 2017 ATA standards was 5.3%, which was significantly higher than those SCH women diagnosed the 2011 ATA standards at 2.1% even when other traditional risk factors were considered. And we also found that SCH women defined by the 2017 ATA standards had increased trends in prevalence of CS (42.1 vs. 36.9%), preterm delivery (53.9 vs. 47.4%), total adverse maternal (69.1 vs. 62.2%), and neonatal outcomes (58.6 vs. 51.1%) than those defined by the 2011 ATA standards, although no statistically significant differences were observed between them. However, the prevalence of other adverse pregnancy outcomes was not significantly different between the two groups.

**Table 4:** Comparisons on the basic characteristics and the incidences of adverse pregnancy outcomes between SCH defined by the 2011 and 2017 ATA standards.

**Variables**	**SCH 2011**	**SCH 2017**	***P-*value**	***P*′ value**	^*^P-value
Maternal age (years)^a^	29 (26–32)	28 (26–32)	0.725	0.807	0.746
Prepregnancy BMI (kg/m^2^)	21.03 ± 2.86	20.64 ± 2.56	0.127	0.144	0.162
Number of pregnancies, n (%)			0.920	0.907	0.363
1	267 (45.6)	72 (47.4)			
2	160 (27.4)	41 (27)			
3+	158 (27)	39 (25.6)			
Number of previous births, n (%)			0.927	0.879	0.709
0	286 (48.9)	73 (48)			
1	285 (48.7)	76 (50)			
2+	14 (2.4)	3 (2)			
Primiparity, n (%)	286 (48.9)	73 (48)	0.850	0.654	0.786
Gestational age (weeks)	38 ± 3	38 ± 3	0.451	0.575	0.613
Gestational weight gain (kg)^a^	12 (10–14)	12 (9–14.25)	0.848	0.675	0.738
**Neonatal**					
Male, n (%)	311 (54)	88 (58.7)	0.305	0.330	0.825
Birth weight (g)	3,275 ± 501	3,206 ± 533	0.138	0.212	0.498
Birth height (cm)	49.7 ± 1.4	49.5 ± 2.1	0.194	0.129	0.279
Apgar scores	9.9 ± 0.7	9.9 ± 1.1	0.372	0.288	0.395
PIH, n (%)	22 (3.8%)	10 (6.6%)	0.129	0.018	0.114
Preeclampsia, n (%)	12 (2.1%)	8 (5.3%)	0.030	0.035	0.037
GDM, n (%)	116 (19.8%)	33 (21.7%)	0.607	0.454	0.464
CS, n (%)	216 (36.9%)	64 (42.1%)	0.241	0.096	0.092
Preterm delivery, n (%)	277 (47.4%)	82 (53.9%)	0.147	0.064	0.076
Postpartum hemorrhage, n (%)	13 (2.2%)	3 (2.0%)	0.851	0.825	0.842
Placenta previa, n (%)	11 (1.9%)	5 (3.3%)	0.288	0.307	0.344
Placenta abruption, n (%)	1 (0.2%)	0 (0%)	0.610	0.676	0.658
Dystocia, n (%)	8 (1.4%)	3 (2.0%)	0.583	0.605	0.607
Low birth weight, n (%)	25 (4.3%)	9 (5.9%)	0.388	0.420	0.371
Preterm birth, n (%)	277 (47.4%)	82 (53.9%)	0.147	0.064	0.076
Macrosomia, n (%)	27 (4.6%)	6 (3.9%)	0.723	0.773	0.804
Fetal distress, n (%)	15 (2.6%)	6 (3.9%)	0.361	0.430	0.434
Fetal asphyxia, n (%)	2 (0.3%)	0 (0%)	0.270	—	—
Fetal deformities, n (%)	2 (0.3%)	0 (0%)	0.470	0.466	0.519
Stillbirth, n (%)	3 (0.5%)	2 (1.3%)	0.283	0.598	0.308
Total adverse maternal outcomes, n (%)	290 (49.6%)	85 (55.9%)	0.163	0.078	0.093
Total adverse neonatal outcomes, n (%)	299 (51.1%)	89 (58.6%)	0.102	0.047	0.058

a *Non-normal distribution of continuous variables*.

*P value, not adjusted*.

*P′ value, adjusted for maternal age, pregestational BMI*.

* *P value, adjusted for maternal age, pregestational BMI, gestational age, number of previous pregnancies, number of previous births, primiparity, and gestational weight gain*.

### Association of Maternal TSH Levels and Adverse Pregnancy Outcomes

Table 5 displays the associations of maternal TSH levels and adverse pregnancy outcomes classified by maternal TSH values. First, except PIH (2.8 vs. 1.5%, OR = 2.99, 95% CI = 1.24–7.23), almost no correlations were observed on the adverse pregnancy outcomes between the normal TSH group (0.27–2.5 mIU/L) and the mildly elevated TSH group (2.5–4.0 mIU/L), after adjustment for potential confounders. Second, compared with the mildly elevated TSH group and the normal TSH group, the significantly elevated TSH group (4.0–10 mIU/L) exhibited remarkably higher incidences and risks of PIH (6.6 vs. 2.8 vs. 1.5%), preeclampsia (5.3 vs. 0.9 vs. 0.5%), CS (42.1 vs. 35.1 vs. 36.4%), preterm delivery (53.9 vs. 45 vs. 48.2%), and total adverse maternal (58.6 vs. 48.5 vs. 52.8%) and neonatal outcomes (69.1 vs. 60 vs. 63.5%). Third, compared with the normal TSH group, the significantly elevated TSH group had significantly increased rates and risks of placenta previa (3.3 vs. 1.3%, OR = 4.79, 95% CI = 1.53–14.93) and low birth weight (5.9 vs. 2.7%, OR = 2.64, 95% CI = 1.14–6.14), whereas no such differences were found when compared with the mildly elevated TSH group. Finally, no statistically significant differences were found in the prevalence and risks of GDM, postpartum hemorrhage, placental abruption, dystocia, macrosomia, fetal distress, fetal asphyxia, fetal deformities, and stillbirth among these three groups.

**Table 5:** Association of maternal TSH with adverse pregnancy outcomes.

**Outcomes**	**Normal TSH group**	**Mildly elevated TSH group**	**Significantly elevated TSH group**	***P*-value**
	**(*n* = 971)**	**(*n* = 433)**	**(*n* = 152)**
PIH, n (%)	15 (1.5%)	12 (2.8%)	10 (6.6%)	
Unadjusted OR (95% CI)	1 (ref)	1.82 (0.84–3.91)	4.49 (1.98–10.18)^*^^+^	0.002
Adjusted OR (95% CI)	1 (ref)	2.99 (1.24–7.23)	8.18 (3.23–20.73)^*^^+^	<0.001
Preeclampsia, n (%)	5 (0.5%)	4 (0.9%)	8 (5.3%)	
Unadjusted OR (95% CI)	1 (ref)	1.80 (0.48–6.74)	10.73 (3.46–33.26)^*^^+^	<0.001
Adjusted OR (95% CI)	1 (ref)	3.37 (0.76–15.04)	18.56 (4.77–72.21)^*^^+^	<0.001
GDM, n (%)	194 (20%)	83 (19.2%)	33 (21.7%)	
Unadjusted OR (95% CI)	1 (ref)	0.95 (0.71–1.27)	1.11 (0.73–1.68)	0.794
Adjusted OR (95% CI)	1 (ref)	1.02 (0.75–1.38)	1.22 (0.79–1.89)	0.668
CS, n (%)	353 (36.4%)	152 (35.1%)	64 (42.1%)	
Unadjusted OR (95% CI)	1 (ref)	0.95 (0.75–1.20)	1.27 (0.90–1.80)	0.298
Adjusted OR (95% CI)	1 (ref)	1.11 (0.85–1.44)	1.74 (1.19–2.54)^*^^+^	0.017
Preterm delivery, n (%)	468 (48.2%)	195 (45%)	82 (53.9%)	
Unadjusted OR (95% CI)	1 (ref)	0.88 (0.70–1.11)	1.26 (0.89–1.77)	0.159
Adjusted OR (95% CI)	1 (ref)	1.01 (0.79–1.30)	1.66 (1.15–2.40)^*^^+^	0.023
Postpartum hemorrhage, n (%)	12 (1.2%)	10 (2.3%)	3 (2.0%)	
Unadjusted OR (95% CI)	1 (ref)	1.89 (0.81–4.41)	1.61 (0.45–5.77)	0.323
Adjusted OR (95% CI)	1 (ref)	1.66 (0.69–4.00)	1.25 (0.34–4.62)	0.530
Placenta previa, n (%)	13 (1.3%)	6 (1.4%)	5 (3.3%)	
Unadjusted OR (95% CI)	1 (ref)	1.04 (0.39–2.74)	2.51 (0.88–7.13)	0.205
Adjusted OR (95% CI)	1 (ref)	1.64 (0.55–4.33)	4.79 (1.53–14.93)^+^	0.026
Placenta abruption, n (%)	1 (0.1%)	1 (0.2%)	0 (0%)	
Unadjusted OR (95% CI)	1 (ref)	2.25 (0.14–35.98)	—	0.849
Adjusted OR (95% CI)	1 (ref)	2.64 (0.12–57.86)	—	0.827
Dystocia, n (%)	14 (1.4%)	5 (1.2%)	3 (2.0%)	
Unadjusted OR (95% CI)	1 (ref)	0.80 (0.29–2.23)	1.38 (0.39–4.85)	0.760
Adjusted OR (95% CI)	1 (ref)	0.78 (0.27–2.24)	1.34 (0.37–4.90)	0.762
Low birth weight, n (%)	26 (2.7%)	16 (3.7%)	9 (5.9%)	
Unadjusted OR (95% CI)	1 (ref)	1.40 (0.74–2.63)	2.29 (1.05–4.98)^+^	0.105
Adjusted OR (95% CI)	1 (ref)	1.65 (0.81–3.36)	2.64 (1.14–6.14)^+^	0.065
Preterm birth, n (%)	468 (48.2%)	195 (45%)	82 (53.9%)	
Unadjusted OR (95% CI)	1 (ref)	0.88 (0.70–1.11)	1.26 (0.89–1.77)	0.159
Adjusted OR (95% CI)	1 (ref)	1.01 (0.79–1.30)	1.66 (1.15–2.40)^*^^+^	0.023
Macrosomia, n (%)	47 (4.8%)	21 (4.8%)	6 (3.9%)	
Unadjusted OR (95% CI)	1 (ref)	1.00 (0.59–1.70)	0.81 (0.34–1.92)	0.886
Adjusted OR (95% CI)	1 (ref)	1.22 (0.49–3.00)	1.19 (0.46–3.06)	0.912
Fetal distress, n (%)	17 (1.8%)	9 (2.1%)	6 (3.9%)	
Unadjusted OR (95% CI)	1 (ref)	1.19 (0.53–2.69)	2.30 (0.89–5.94)	0.224
Adjusted OR (95% CI)	1 (ref)	1.40 (0.58–3.35)	2.58 (0.95–7.03)	0.178
Fetal asphyxia, n (%)	3 (0.3%)	2 (0.5%)	0 (0%)	
Unadjusted OR (95% CI)	1 (ref)	1.50 (0.25–8.99)	—	0.907
Adjusted OR (95% CI)	1 (ref)	1.16 (0.11–12.40)	—	0.993
Fetal deformities, n (%)	12 (1.2%)	2 (0.5%)	0 (0%)	
Unadjusted OR (95% CI)	1 (ref)	0.37 (0.08–1.66)	—	0.432
Adjusted OR (95% CI)	1 (ref)	0.82 (0.11–6.40)	—	0.982
Stillbirth, n (%)	9 (0.9%)	1 (0.2%)	2 (1.3%)	
Unadjusted OR (95% CI)	1 (ref)	0.25 (0.03–1.96)	1.43 (0.31–6.66)	0.341
Adjusted OR (95% CI)	1 (ref)	0.31 (0.03–3.05)	1.37 (0.15–12.60)	0.548
Total adverse maternal outcomes, n (%)	481 (49.5%)	205 (47.3%)	85 (55.9%)	
Unadjusted OR (95% CI)	1 (ref)	0.92 (0.73–1.15)	1.29 (0.92–1.82)	0.192
Adjusted OR (95% CI)	1 (ref)	1.04 (0.81–1.33)	1.63 (1.13–2.36)^*^^+^	0.032
Total adverse neonatal outcomes, n (%)	617 (63.5%)	260 (60%)	105 (69.1%)	
Unadjusted OR (95% CI)	1 (ref)	0.84 (0.67–1.06)	1.26 (0.89–1.78)	0.083
Adjusted OR (95% CI)	1 (ref)	1.03 (0.80–1.33)	1.72 (1.19–2.50)^*^^+^	0.014

*Adjusted OR: adjusted for maternal age, pregestational BMI, number of pregnancies, number of previous births, primiparity, gestational age, and gestational weight gain*.

* *P < 0.05 (2.5 ≤ TSH <4.0 mIU/L vs. 4 ≤ TSH <10 mIU/L)*.

+ *P < 0.05 (0.29 ≤ TSH <2.5 mIU/L vs. 4 ≤ TSH <10 mIU/L)*.

## Discussion

The extremely high circulating human chorionic gonadotropin levels along with the increased synthesis of thyroxine-binding globulin in the first trimester can trigger maternal thyroid hormone alteration and result in a variety of thyroid disorders (18). Some studies have discovered the adverse effects of maternal abnormal thyroid disorders on pregnancy outcomes (1, 19, 20). However, the associations between maternal SCH in pregnancy and adverse obstetrical outcomes still remain unclear, which also obtained increasing concerns from endocrinologists and gynecologists recently. Therefore, we carried out the present study to evaluate and compare the effects of maternal SCH diagnosed by the 2011 and 2017 ATA guidelines on adverse perinatal outcomes during the early pregnancy in TPOAb-negative pregnant women. To the best of our knowledge, this is the first time to systematically compare the prevalence and risks of adverse maternal and fetal outcomes through different SCH diagnostic criteria in healthy pregnant population.

In the current study, SCH pregnant women diagnosed by both the 2011 and 2017 ATA guidelines successively exhibited the significantly higher incidences and risks of PIH and preeclampsia in the first trimester compared with those euthyroid pregnant women. More interestingly, we found the incidence of preeclampsia but not PIH was significantly higher in SCH women identified by the 2017 ATA guidelines than that in those SCH women diagnosed by the 2011 ATA guidelines after controlling for various confounding factors, which indicated that the elevated TSH concentration may be related to the severity of gestational hypertension. Consistently with us, Wu et al. (21) found that Chinese pregnant women diagnosed with SCH in the first and second trimesters were more likely to develop hypertensive disorders of pregnancy during the remainder of their pregnancy. Similar with us, another population-based study involving 24,883 Hispanic women demonstrated that the incidence of hypertension in the SCH group identified by TSH levels more than 4.13 mIU/L was 10.9%, which was significantly greater than that in the euthyroid group at 6.2% (4), while different from us and Wu et al. (21). There was the only remaining significant association between SCH and severe preeclampsia after adjusting for confounding factors in their study. A possible explanation for the difference was that the latter did not consider the screening time for thyroid function and TPOAb status. The mechanism of remarkable link between SCH and pregnancy-associated hypertension had been partially clarified by a molecular research, which demonstrated that individuals with SCH were characterized by impaired endothelium-related vasodilation due to the reduction of nitric oxide availability, and this alteration could be reversed by LT4 supplementation (22).

Another interesting and unexpected finding were that SCH women identified by the 2017 ATA criteria exhibited significantly higher risks of developing CS, placenta previa, preterm labor, and overall adverse pregnancy outcomes when compared with euthyroid women, whereas no such remarkable associations were observed in women with SCH diagnosed by the 2011 ATA criteria. The risks of above pregnancy complications in relation to maternal SCH had been previously studied with contradictory conclusions that several trails showed positive associations of SCH with adverse pregnancy outcomes (5, 23), whereas others did not demonstrate increased risks between them (7, 21). For example, Su et al. (5) found that SCH defined as TSH greater than the 95th percentile (at least 3.77 mIU/L) in the first 20 weeks of pregnancy may result in 3.32 (1.22–9.05)-fold risk of preterm delivery. But Wu et al. (21) reported that no statistical difference in preterm labor was noted between SCH women (TSH greater than the 95th percentile) and euthyroid women in the first trimester. Therefore, different cutoffs and definitions of SCH leading to different perinatal outcomes among the same crowd in our present study may well-explain these discrepancies. Consistent with our findings, Nazarpour et al. (24) found that LT4 could precisely decrease the risk of preterm delivery using the TSH cutoff ≥4.0 mIU/L, whereas there was no beneficial effect of LT4 therapy in reducing this complication in SCH women with negative TPOAb with a TSH cutoff point of 2.5 mIU/L.

Additionally, we also found that women with SCH diagnosed by the 2011 ATA standards were at significantly greater risk of low-birth-weight delivery, same as the results reported by Leung et al. (25). But no such an association was found among women with SCH diagnosed by the 2017 ATA standards; even the incidence of low birth weight among women with SCH diagnosed by the 2011 ATA standards was almost two times higher than that among euthyroid women, which suggested that SCH diagnosed by the 2011 ATA standards could predict a small portion of adverse pregnancy outcomes, and whether the interventions were needed to reduce these adverse events is still worth further study.

However, we did not find that SCH diagnosed by either 2011 or 2017 ATA guidelines increased the risk of GDM, postpartum hemorrhage, placental abruption, dystocia, macrosomia, fetal asphyxia, fetal distress, fetal deformities, and stillbirth development. Tudela et al. (26) documented that the likelihood of GDM remarkably increased with elevated TSH level, but similar with us, no significant association was observed in patients with SCH after adjustment for confounding factors. Likewise, Krassas et al. (27) and Matalon et al. (28) revealed that no association was seen between SCH and postpartum hemorrhage, although Feldthusen et al. (23) observed the notably higher prevalence of postpartum hemorrhages in women with SCH than that in euthyroid women in the first trimester without adjusting the interfering factors such as BMI. In addition, the significant association of SCH with placental abruption was observed only in one study (29), whereas this was not found in our study and others (30, 31). Chen et al. (31) also displayed that no significant differences were noted in GDM, placental abruption, fetal distress, stillbirth, and malformation between the SCH and euthyroid groups in the first trimester. Wu et al. (21) also reported there were no statistical differences in GDM, fetal distress, perinatal mortality, and birth weight more than 4,000 g between the SCH and euthyroid groups in the first trimester after controlling for confounding parameters.

Nowadays, there is a remaining controversial issue regarding whether a mildly elevated TSH concentration is related to obstetric complications. Some studies have reported that a mildly elevated TSH positively correlated with multiple possible gestational complications, such as spontaneous abortion, GDM (26, 32). For example, a prospective study performed in TPOAb-negative southern Italy pregnant women by Negro et al. (32) found a significantly higher rate of spontaneous fetal loss in pregnant women with TSH between 2.5 and 5 mIU/L in the first trimester compare with those with TSH <2.5 mIU/L. However, Li et al. (14) and Rosario et al. (13) argued that there were no associations between TPOAb-negative women with TSH concentration between 2.5 and 4.0 mIU/L during their first trimesters and the incidences of recorded adverse pregnancy outcomes without controlling for other confounding factors. Consistent with the studies by Li et al. (14) and Rosario et al. (13), before adjustment for potential risk factors, our results showed that no statistical significance was observed between a mildly elevated TSH concentration and a variety of obstetric complications in Chinese TPOAb-negative pregnant women. However, we found a significant association between a mildly elevated TSH and PIH after adjustment for other factors, which suggested that these additional variables may act as compounding factors. When we adjusted them, the association between a mildly elevated TSH and PIH was obvious. Therefore, our current study further provided the evidence that a mildly elevated TSH concentration only remarkably related to a small portion of adverse pregnancy outcomes.

On the contrary, we found that pregnant women with TSH >4.0 mIU/L had remarkably higher incidences and risks of PIH, preeclampsia, CS, preterm delivery, and total adverse maternal and neonatal outcomes when compared with those with TSH <2.5 mIU/L and TSH between 2.5 and 4 mIU/L. And women with TSH >4.0 mIU/L exhibited significantly higher rate and risks of placenta previa and low-birth-weight delivery compared with those with TSH <2.5 mIU/L. These results indicated that pregnant women with TSH > 4.0 mIU/L remarkably related to the most portion of adverse pregnancy outcomes. In line with us, Arbib et al. (33) displayed that women with TSH 2.5–4.0 mIU/L and >4.0 mIU/L successively had 1.81- and 2.33-fold risk of preterm delivery even when other traditional risk factors were considered. A study by Carty et al. (8) also demonstrated that women with TSH >5 mU/L delivered infants with lower birth weight than those with TSH <2.5 mU/L. However, there were no differences in preeclampsia, CS, preterm delivery in their study, which was different from us and may be explained by the facts that pregnant women in our study had higher prevalence of CS than those in the study by Carty et al. (8). Additionally, women with TSH level of > 5 mU/L in the study of Carty et al. included some women with FT4 levels below the non-pregnant reference range (9–21 pmol/L). In summary, our above results further fortify and enhance the current body of evidence suggesting that SCH diagnosed by the standards of 2017 ATA were apt to screen high-risk Chinese pregnant women compared with the 2011 ATA guidelines.

In addition, our findings were obtained from Chinese pregnant women with negative TPOAb, which provided robust evidence to support the opinion from Rotondi et al. (34). They thought the 2017 ATA guidelines overemphasized thyroid autoantibodies status and recommended treatment with LT4 in pregnant women with TSH levels ranging between the upper limit of the reference range and 10.0 μIU/mL independent from their thyroid antibody status. And our present study also further confirmed that some detrimental effects of maternal SCH may be actually present even in women with negative tests for TPOAb. This could be explained by the facts that thyroid antibodies may be falsely negative during gestation and that chronic autoimmune thyroiditis with serum negative thyroid autoantibodies is a well-known clinical entity even outside pregnancy (35, 36).

Our study has some limitations. First, the study population of these findings is Chinese, and thus more studies may need to verify whether these findings may be generalized to other populations. Second, long-term outcomes of the offspring like IQ are not considered in our study as we only make research to examine obstetric outcomes. Third, we did not consider the effect of thyroglobulin antibody on pregnancy outcome and not assess the effects of SCH in different trimesters on maternal and perinatal outcomes.

## Conclusions

In general, our results extended and enhanced previous work and indicated that maternal SCH diagnosed by the 2017 ATA guidelines increased the risks of multiple adverse pregnancy outcomes, whereas maternal SCH diagnosed by the 2011 ATA guidelines was associated with only a small portion of adverse pregnancy outcomes in Chinese healthy pregnancy women with negative TPOAb during early pregnancy. Moreover, our current study further provided the evidence that a mildly elevated TSH concentration remarkably related to PIH even after adjustment. Our findings suggest that the 2017 ATA guidelines could be more applicable to Chinese pregnant women to screen the effects of SCH on the majority of detrimental maternal and neonatal outcomes, whereas the 2011 ATA guidelines were more suitable to evaluate SCH effects on a small portion of adverse pregnancy outcomes, such as PIH, preeclampsia, and low birth weight.

## Data Availability Statement

The raw data supporting the conclusions of this article will be made available by the authors, without undue reservation.

## Ethics Statement

The studies involving human participants were reviewed and approved by Shanghai Jiao-Tong University Affiliated Sixth People's Hospital. The patients/participants provided their written informed consent to participate in this study.

## Author Contributions

L-XL designed the study, reviewed, and edited the manuscript. LM conducted the obstetric and surgical clinical practice. YZ, T-PY, and J-FK collected samples and clinical data. YL performed statistical analysis. M-FL and Q-MF worked together and wrote the manuscript. All authors revised the manuscript and approved the final manuscript.

## Conflict of Interest

The authors declare that the research was conducted in the absence of any commercial or financial relationships that could be construed as a potential conflict of interest.
