# Supplementary material for: Effects of Maternal Subclinical Hypothyroidism in Early Pregnancy Diagnosed by Different Criteria on Adverse Perinatal Outcomes in Chinese Women With Negative TPOAb
Source: Front Endocrinol (Lausanne). 2020 Oct 8;11:580380. doi: 10.3389/fendo.2020.580380 (PMC7578396; doi:10.3389/fendo.2020.580380)
Supplement: Supplementary file 1 [file Table_1.docx]

**Table S1 The diagnostic criteria of pregnancy outcomes**

| **Outcomes** | **Diagnostic criteria** |
| --- | --- |
| PIH | PIH was diagnosed when new-onset hypertension (SBP ≥ 140 mmHg and /or DBP ≥ 90 mmHg), occurring after 20 weeks' gestation. |
| Preeclampsia | Preeclampsia was defined as PIH associated with proteinuria ≥ 0.3 g/24 h after 20 weeks’ gestation. |
| GDM | GDM was diagnosed when fasting blood glucose (FBG) ≥ 5.1 mmol/L during the first trimester or when one of the 75 g OGTT conditions met: FBG ≥ 5.1 mmol/L; 1 h after glucose load ≥ 10.0 mmol/L; 2 h after glucose load ≥ 8.5 mmol/L. |
| CS | CS was recorded by the midwives who participated in labor. |
| Preterm delivery | Preterm delivery was identified when the delivery occurred before 37 weeks’ gestation. |
| Postpartum hemorrhage | Postpartum hemorrhage was recognized when more than 500 ml or 1,000 ml of blood loss within the first 24 hours after childbirth. |
| Placenta previa | Placenta previa was the placenta grew in the lowest part of the womb (uterus) and covered all or part of the opening to the cervix. |
| Placenta abruption | Placental abruption occurred when the normally located placenta completely or partially separated from the uterine site before the birth of fetus. |
| Dystocia | Dystocia referred to abnormal or difficult childbirth or labor diagnosed by an obstetrician. |
| Total adverse maternal outcomes | One or more above complications resulted in the total adverse maternal outcomes. |
| Low birth weight | low birth weight < 2500g. |
| Preterm birth | Preterm delivery was identified when the delivery occurred before 37 weeks’ gestation. |
| Macrosomia | Macrosomia: ≥ 4000g |
| Fetal distress | Fetal distress was recognized when the symptoms of fetal hypoxia occurred, including fetal bradycardia, serious variable decelerations and lasting late decelerations. |
| Fetal asphyxia | Fetal asphyxia as identified when Apgar score < 7 at 1 min. |
| Fetal deformities | Fetal deformities were diagnosed either by ultrasonography in pregnancy or during the neonatal period. |
| Stillbirth | Stillbirth was referred as a baby born with no signs of life with gestation period of ≥ 28 weeks. |
| Total adverse neonatal outcomes | One or more above characters generated the total adverse neonatal outcomes. |
